# Effect of Nanoclay Particles on the Performance of High-Density Polyethylene-Modified Asphalt Concrete Mixture

**DOI:** 10.3390/polym13030434

**Published:** 2021-01-29

**Authors:** Ghada S. Moussa, Ashraf Abdel-Raheem, Talaat Abdel-Wahed

**Affiliations:** 1 Civil Engineering Department, Assiut University, Assiut 71515, Egypt; 2 Civil Engineering Department, Sohag University, Sohag 82524, Egypt; ashrf_salah@eng.sohag.edu.eg (A.A.-R.); talaat_ali@eng.sohag.edu.eg (T.A.-W.)

**Keywords:** high-density polyethylene, nanoclay, polymer nanocomposite, asphalt binder modification, modified AC mixture, storage stability, stiffness, moisture damage, stripping, rutting

## Abstract

Utilizing polymers for asphalt concrete (AC) mixture modification has many drawbacks that hinder its wide implementations for roadway construction. Recently, research on employing complementary materials, such as nanomaterials, to balance negative impacts of polymers while enhancing the AC mixture’s performance has received great attention. This study aimed to investigate the effect of incorporating nanoclay (NC) particles on the performance of a high-density polyethylene (HDPE)-modified AC mixture. A 60/70 asphalt binder was first modified with HDPE, and then NC particles were gradually added at a concentration of 1–4% by weight of the asphalt binder. The binders’ physical characteristics, storage stability, and chemical change were scrutinized. AC mixture performance, including pseudo-stiffness, moisture damage resistance, stripping susceptibility, and rutting tendency, was investigated. A statistical analysis on the experimental results was conducted using Kruskal–Wallis and Dunn tests. Test results showed that employing NC/HDPE significantly increased penetration index and thereby enhanced binder temperature sensitivity. Moreover, it prevented oxidation action and separation and, therefore, enhanced binder storage stability. Furthermore, incorporating NC amplified pseudo-stiffness and significantly improved resistance against moisture damage and stripping of HDPE-modified mixtures. Moreover, it improved both elastic (recoverable) and plastic (unrecoverable) deformations of mixtures. The most satisfactory results were attained when incorporating 3% of NC.

## 1. Introduction

With the tremendous evolution in traffic and adverse climatic conditions, techniques to increase pavement service life while reducing maintenance need became an urge. Therefore, utilizing modified asphalt concrete (AC) mixtures for roadway construction has gained remarkable attention due to their superior performance compared to the traditional ones [1,2]. Polymers are among the most prevalent asphalt modifiers due to their ability to enhance AC mixture stiffness, visco-elastic behaviour, and durability at various temperatures [3,4,5,6,7]. Thus, polymer-modified AC mixtures exhibited a noticeable resistance to thermal cracking, rutting, as well as water and fatigue damages. Various types of polymers, including styrene-butadiene-styrene (SBS), styrene-butadiene-rubber (SBR), styrene-ethylene/butylene-styrene (SEBS), ethyl-vinyl-acetate (EVA), polypropylene (PP), polyethylene (PE), low-density polyethylene (LDPE), and high-density polyethylene (HDPE) have been incorporated for enhancing asphalt performance. Nevertheless, there are some drawbacks of utilizing polymers as asphalt modifiers, such as poor storage-stability due to asphalt/polymer density variation, oxidation, and underprivileged adhesion [1,8,9]. Therefore, various attempts to overcome such limitations by implementing a complementary material, such as nanomaterials, that can balance the negative impact of polymers while enhancing the AC mixture performance have been investigated. 

Nanomaterials have recently received prodigious attention as asphalt additives due to their eminent characteristics comprising large surface area, high dispersion capability, and high chemical purity [10,11,12,13]. Research on implementing nanomaterials in combination with polymers for pavement performance enhancement has turned into an emerging interest area, where adding nanomaterials to polymer-modified asphalt not only bridges the polymer/asphalt gap but also magnifies the polymer’s effect on enhancing the asphalt performance, particularly at high temperatures [9,14,15]. The most common types of nanomaterials that have been employed in combination with polymers for asphalt modifications include nanoclay (NC), nanosilica (NS), carbon-nanotubes (CNT), and nanolime [13,16]. Table 1 summarizes the relevant polymer–nanocomposite literature studies.

Based on the literature, incorporating NC particles with styrene-butadiene-styrene (SBS) polymer improved binder physical and rheological properties [17,18,19,20,21,22,23,24], aging characteristics [25], storage stability [21,22,26], fatigue cracking resistance [6,27], and rutting resistance [23,24]. Ren et al., 2020 investigated the effect of NC on the physical and rheological properties and the storage stability of three polymer-modified binders using; crumb rubber (CRM), styrene-butadiene-styrene (SBS), and styrene-butadiene-rubber (SBR) [28]. Based on their results, the addition of NC slightly increased the viscosity but did not affect the three polymer-modified binders’ rheological properties. While the addition of NC caused a significant improvement in the CRM-modified binder’s storage stability, it had an insignificant effect on that of the SBS- and SBR-modified binders. In another study conducted by Amini et al., 2017, the effect of a composite of NC and SBR with different ratios on the binder and AC mixture performance was evaluated [29]. They reported an improvement in the binder’s storage stability and in the AC mixture’s moisture sensitivity with the addition of NC/SBR composite. Moreover, two studies conducted by Zapién-Castillo et al., 2016 and Azarhoosh and Koohmishi 2020 considered the addition of NC/SEBS composites with different ratios [30,31]. Zapién-Castillo et al. reported an enhancement in the binder’s physical and rheological characteristics and storage stability as well [30]. In comparison, Azarhoosh and Koohmishi concluded improvements in the modified mixtures’ rutting potential compared with the control one [31]. In another study conducted by Siddig et al., 2018, the effect of incorporating NC, EVA, and NC/EVA on the physical and rheological properties of binders was investigated [32]. Their results revealed that incorporating NC and EVA separately or in combination remarkably improved the binders’ characteristics. However, the NC/EVA-modified binder outperformed the control, NC-, and EVA-modified binders in terms of the high-temperature performance. Additionally, Ghaempour et al., 2015 reported an improvement in binder rheological characteristics when incorporating NC/EVA [33]. When combining NS with SBS for binder modification, Yusoff et al., 2014, Ghasemi et al., 2012, and Shafabakhsh et al., 2019 investigated the NS/SBS effect on the binder and mixture behaviour [5,34,35]. Yusoff et al. concluded that adding NS/SBS improved the moisture susceptibility, fatigue, and rutting behaviour of the asphalt mixtures. The results of the study by Ghasemi et al. implied that incorporating NS/SBS enhanced the physical and mechanical characteristics of the binder and the mixture. Shafabakhsh et al. reported a significant improvement in the fatigue-life of both the binder and mixture compared with the control ones. Moreover, Bala et al., 2018 studied the effect of the addition of NS on polypropylene (PP)-modified asphalt mixture [7]. Their results showed that incorporating NS enhanced fatigue characteristics and strength of the PP-modified asphalt mixture. Goli et al., 2017 investigated the addition of CNT on the performance of the SBS-modified asphalt binder [36]. Their results showed that adding CNT enhanced the temperature susceptibility, physical, and rheological characteristics of the SBS-modified asphalt binder. Another study revealed a positive effect of incorporating nano-Al_2_O_3_ on the storage stability and high-temperature behaviour of an SBS-modified asphalt binder [37]. Moreover, two studies evaluated the effect of the composition of two nanomaterials (NC and nanolime) with SBS on the binder’s rutting resistance [37,38]. Both studies reported an improvement in the binder’s rutting parameters when incorporating NC/nanolime/SBS composite. On utilizing nanomaterials with two types of polymers as a composite, Sun et al., 2017 and Mansourian et al., 2019 investigated the effect of NS/SBR/PE and NC/HDPE/EVA composites, respectively, on the asphalt binder’s behaviour [14,39]. Sun et al., 2017 concluded an improvement in the high- and low-temperature performance compared with the unmodified- and SBS-modified binders. Mansourian et al., 2019 reported that the addition of NC/HDPE/EVA composites enhanced the low-temperature performance and rutting resistance of the asphalt binder. 

To this end, most of the previous studies dedicated to exploring polymer/nanocomposites for asphalt modification focused on incorporating thermoplastic-elastomer polymers, such as SBS and SBR, which are relatively expensive compared with other elastomer polymers, such as HDPE [29,40,41,42]. Moreover, most previous research efforts on polymer/nanocomposite have investigated the performance of the modified asphalt binder; however, a lack of research has investigated the modified AC mixture’s performance. However, investigating the performance of the modified AC mixture is needed since it is directly related to the overall pavement behaviour under traffic loading and environmental conditions. The current study attempts to fill this gap via experimentally investigating the effect of incorporating NC particles on the performance of the HDPE-modified asphalt binder and mixture as well. 

## 2. Materials and Methods

### 2.1. Materials

#### 2.1.1. Binder

In this study, a 60/70 penetration grade base asphalt binder produced by Suez Petroleum Company (Suez, Egypt), was utilized for the modified binder preparation. Its physical and mechanical characteristics are listed in Table 2. 

#### 2.1.2. Aggregate

The utilized aggregate for both the conventional (unmodified) and modified AC mixture comprises crushed dolomite stones with nominal maximum aggregate size (NMAS) of 19-mm, and siliceous sand as coarse and fine aggregate, respectively. Moreover, limestone dust was employed as a mineral filler. 

#### 2.1.3. Additives

**Figure 1:**
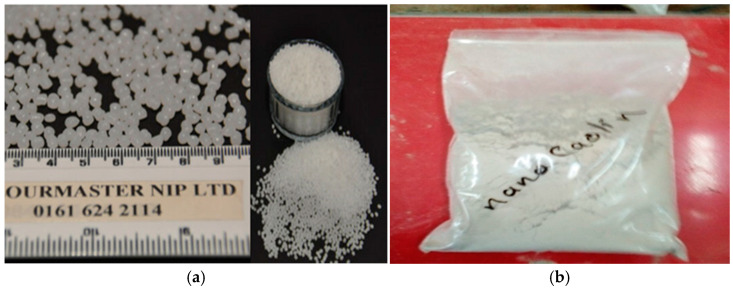
Utilized additives; (**a**) high-density polyethylene (HDPE) resin; (**b**) nanoclay (NC) powder.

In this study, two types of additives, high-density polyethylene (HDPE) resin and NC powder, were utilized for binder modification, as displayed in Figure 1a,b, respectively. The physical and mechanical properties data sheet of the HDPE resin is presented in Table 3. The employed NC modifier was a high purity Kaolin NC (Kaolinite-1Ad) with physical and chemical specifications as listed in Table 4.

### 2.2. Methods

As the main objective of this work was to experimentally investigate the effect of NC particles on the performance of the HDPE-modified AC mixture, five phases were considered in the experimental methodology. In the first phase, the selected materials were employed for the preparation of HDPE- and NC/HDPE-modified binders. The second phase considered AC mixtures’ design and preparation. The third phase included binders’ physical characteristics, storage stability, chemical change, and microstructural investigation tests. The fourth phase experimentally evaluated both conventional and modified AC mixtures’ stiffness, moisture damage resistance, stripping susceptibility, and rutting resistance through Marshall, immersion–compression, double punching, and static creep tests, respectively. All experimental tests in the third and fourth phases were performed on three replicates for each group of AC binders and mixtures. Finally, the fifth phase statistically investigated the experimental results values obtained from phases three and four. 

#### 2.2.1. NC Particles Characterization 

**Figure 2:**
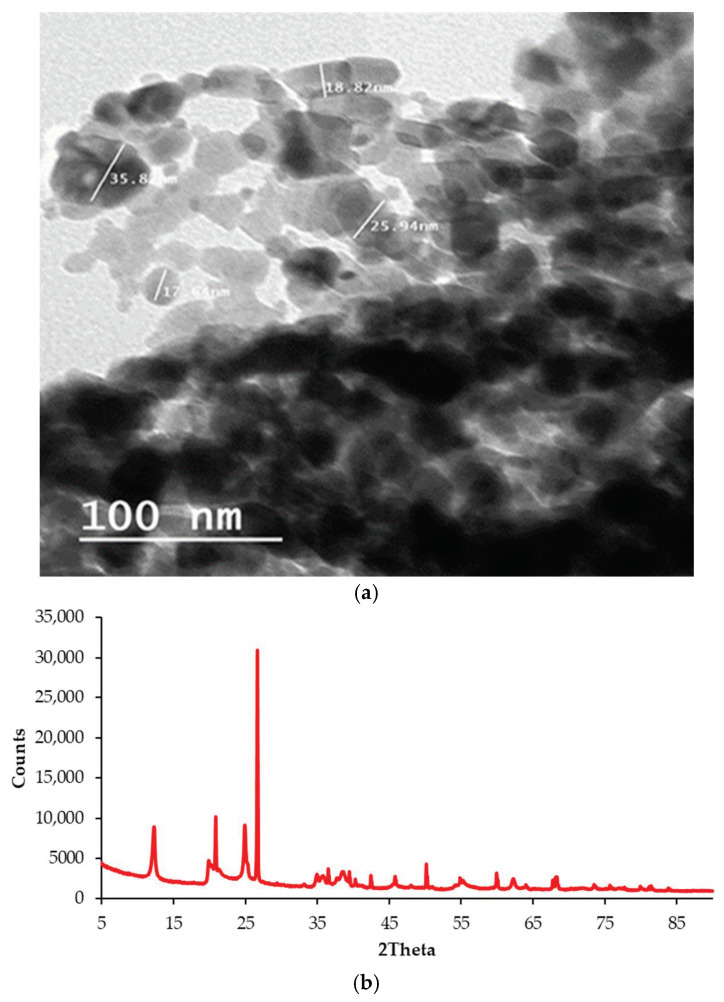
Characteristics of employed NC: (**a**) NC under SEM; (**b**) XRD spectrum of NC powders.

For verification of NC particles’ size-distribution, Scanning Electron Microscopy (SEM) was utilized. Moreover, for identification of the distance between NC interlayers, X-ray diffraction (XRD) analysis was conducted, in which an X-ray diffractometer using Cu-Kα radiation with a wavelength of 1.541 Å (λ), and a scan range (2θ) of 5.007° to 89.9757° with a step size of 0.026°, was utilized. Figure 2a,b present an image of NC particles under SEM and the XRD spectrum, respectively.

The XRD spectrum of NC can be used for estimating the interlayer spacing using the following Equation:

(1) 2dsinθ=λ

where d is the interlay distance, λ is the diffractometer’s wavelength, and θ is the diffraction angle value at which the first strong peak in the XRD spectrum is attained.

XRD spectrum of NC showed a strong diffraction peak at 2θ = 26.6137° with an interlay spacing (d) of 3.34852 Å.

#### 2.2.2. Sample Preparation 

In the sample preparation phase, HDPE- and NC/HDPE-modified binders were prepared. In the preparation of the HDPE-modified binder (P), 4% of HDPE by weight of the base binder was mixed with the base binder [46]. For NC/HDPE- modified binder preparation, NC particles with concentrations of 1%, 2%, 3%, and 4% by weight of the base binder were added to the HDPE-modified binder. NC/HDPE-modified samples were recorded as PC1, PC2, PC3, and PC4.

**Figure 3:**
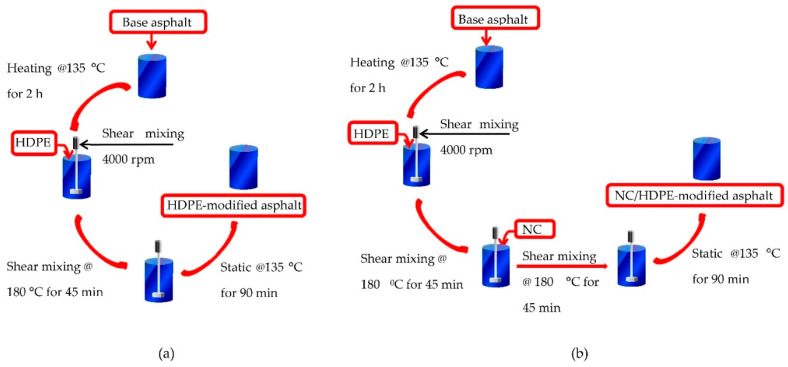
Schematic representations of modified binder preparation: (**a**) HDPE-modified binder and (**b**) NC/HDPE-modified binder.

Figure 3 illustrates the preparation process of modified asphalt binders. First, for removing any trapped moisture in the base asphalt, it was heated to a temperature of 135 °C for a 2 h period. Both HDPE- and NC/HDPE-modified binders were prepared using a high-shear mixer with a 4000 rpm rotational speed at 180 °C. For HDPE-modified binder preparation, HDPE was mixed with the base binder for 45 min, as shown in Figure 3a. However, in the NC/HDPE-modified binder preparation, first, HDPE was completely dissolved in the base binder (45 min shear mixing), then NC particles were gradually added and mixed for 45 min as illustrated in Figure 3b. Finally, all modified binders were set for a 90 min period at 180 °C in a static condition. 

#### 2.2.3. Mixture Design and Preparation

**Figure 4:**
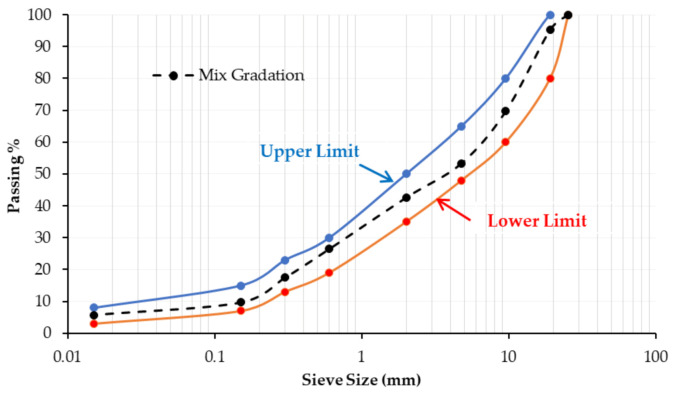
Gradation curves for the conventional AC mixture and specifications.

For the conventional AC mixture design and the optimal asphalt content (OAC) estimation, the Marshall Mix design method (ASTM D 1559) was implemented. The gradation curves for both the conventional AC mixture and the dense-graded AC wearing course (4C) limits, following the Egyptian standard specifications, are depicted in Figure 4 [47]. According to Marshall Design criteria, the OAC for the conventional AC mixture was estimated to be 5%, and all modified AC mixtures were prepared with this value accordingly. Table 5 lists Marshall Parameters for the conventional mixture with the OAC (5%) and their corresponding specification limits. 

For HDPE- and NC/HDPE-modified AC mixture preparation, both aggregate and modified binders were heated (at 180 °C) and mixed thoroughly using a laboratory mixer. 

#### 2.2.4. Binder Evaluation Tests

- Physical characteristics of binders:

The main physical characteristics tests for modified binders were conducted comprising a penetration depth test (ASTM D5), softening point test (ASTM D36), and rotational viscosity test (ASTM D2171), and then their results were compared with those of the base (unmodified) binder. 

While the penetration depth test (ASTM D5) was conducted to evaluate the binder’s hardness, the softening point test (ASTM D36) was executed to appraise the consistency and flow of the binder. Moreover, for assessing the binder’s temperature sensitivity, the penetration index (PI) values were estimated using both penetration depth and softening point values as presented in Equation (2) [48]. A higher PI value reveals lower thermal sensitivity. The recommended PI value for road pavement construction should be in a range of −2 to +2 [48,49].

(2) $$\mathrm{PI}=\frac{1952-500\log P_{25\;}-20T_{R\&B}}{50{\mathrm{logP}}_{25}-T_{R\&B}-120}$$

where P_25_ and T_R&B_ are the penetration value at 25 °C (in 0.1 mm increments) and softening point temperature (in °C), respectively. 

On the other hand, for evaluating the asphalt binder workability, the rotational viscosity (RV) test (ASTM D4402) was conducted. RV, also known as Brookfield viscosity, of the asphalt binder was measured at 135 °C [50].

- Storage stability of binders:

The stability of storage test based on ASTM D7173 was performed to assess the storage ability of modified binders and the phase-separation between bitumen and additives at high temperatures [32,33]. The modified binders were poured into aluminium tubes and kept in upright positions for 5 h at 163 °C, then tubes were allowed to cool in a freezer for 5 h at −10 °C. Afterward, each tube was divided equally into three parts. Then, the difference between the softening point value of the upper- and lower-part was recorded. The softening point’s difference (SP_diff_) between the top and bottom parts should be less than 2.5 °C to ensure acceptable storage stability (ASTM D5892). Moreover, a lower SP_diff_ value reveals a higher storage stability [28,37].

- Chemical change investigation of binders:

The Fourier Transform Infrared Spectroscopy (FTIR) characterization technique was carried out to investigate the chemical structure and molecular-level interactions of asphalt cement before and after adding additives. FTIR is a reliable, widely-used technique that is able to analyse modification mechanisms and recognize chemical changes in the functional groups within asphalt materials [12,50]. Characterization of chemical change and molecular-level interactions plays a critical role in the storage stability and mechanical properties of the asphalt cement [51]. In this study, the Thermo-Scientific Nicolet-iS10 FTIR Spectrometer, with a spectra (band) range of 4000–400 cm^−1^, was utilized. The resulting signal characterizes a molecular fingerprint for the tested material, in which transmittance values at specific wavenumbers are referred to as peaks, and they indicate bonds within the material’s chemical structure [52].

- Microstructural investigation of binders:

The microstructure of the base and modified asphalt binders was investigated using a scanning electron microscope (SEM) for assessing the binder’s homogeneity and the quality of dispersal of additives. The microstructural morphology images of the base, HDPE-, and NC/HDPE-modified asphalt binders were obtained using SEM.

#### 2.2.5. Mixture Evaluation Tests

- Stiffness of mixtures:

For evaluating the mixtures’ stiffness (rigidity), Marshall Quotient (MQ) value was obtained using the Marshall Test results (ASTM D 6927). MQ is a well-known numerical indicator that represents the AC mixture’s stiffness (pseudo-stiffness) and its resistance to shear stress and consequently rutting tendency [37,53,54,55]. According to ASTM D 6927, for each mixture, three cylindrical specimens (64mm—height and 102 mm—diameter) were immersed in water for a 30 min period at 60 °C just before testing. Then, each specimen was laterally loaded in the Marshall testing machine, with a loading rate of 50.8 mm/min, until the specimen’s failure, i.e., reaching the maximum load. The maximum load (in KN) and its corresponding specimen’s deformation (in mm) were recorded as Marshall stability and flow, respectively (ASTM D 6927). For each mixture, the average value of Marshall stability and flow for the three replicates (specimens) was determined. Consequently, for quantifying the asphalt mixture stiffness (rigidity), Marshall Quotient (MQ) value was calculated by dividing the Marshall stability (maximum load) by its corresponding flow (deformation) of the asphalt mixture. Higher MQ values imply stiffer AC mixtures [56].

- Moisture susceptibility of mixtures:

The reduction in asphalt/aggregate adhesion due to moisture infiltration is a well-known phenomenon called “moisture susceptibility” [57]. The moisture susceptibility phenomena, unfortunately, can lead to various pavement distresses, such as cracking, potholes, bleeding, and shoving, and consequently shortening the pavement service-life [34]. For investigating moisture susceptibility of conventional and modified AC mixtures, an immersion–compression test was conducted in accordance with ASTM D 1075 and AASHTO T165.

According to ASTM D 1075, two sets of samples (dry and immersed) were prepared for each mixture. For the dry-set, three samples were stored in an air bath at 25 °C for at least 4 h then tested to determine the compressive strength (CS_Dry_), according to ASTM D 1074. On the other hand, for the immersed-set, three samples were immersed in a water bath at 60 °C for a 24 h period then stored in another water bath at 25 °C for 2 h before they were tested for the compressive strength determination (CS_Immersed_). The compressive strength of both dry and immersed samples was calculated as in Equation (3). 

A numerical index of the AC mixture’s resistance to the moisture effect, called the index of retained strength (IRS), was calculated according to Equation (4) (ASTM D 1074). The IRS value for each mixture was calculated by dividing the average retained compressive strength (immerged) over the average of freshly moulded compressive strength (dry). Based on ASTM D 1074 specifications, the IRS value has to be more than 70%.

(3) $$\mathrm{CS}=\frac{4P}{{\pi D}^{2}}$$

where CS is the compressive strength (Kg/cm^2^), P is the maximum applied compressive load (kg), and D is the sample diameter (cm).

(4) $$\mathrm{IRS}=\frac{{\mathrm{CS}}_{\mathrm{Immersed}}}{{\mathrm{CS}}_{\mathrm{Dry}}}*100$$

where CS_Dry_ and CS_Immersed_ are the average compressive strength of dry and immersed samples, respectively.

- Stripping susceptibility of mixtures:

A double punching (DP) test was carried out in order to assess the stripping susceptibility (deboning) of asphalt from aggregates in the AC mixture [56,57]. The punching strength can be used as an indicator of the action of shear resistance within the AC mixture (i.e., the stripping behaviour between aggregate and binder). In the DP test, asphalt concrete cylindrical specimens (64 mm—height and 102 mm—diameter) were put in a water bath at 60 °C for a 30 min period just before testing. Then, each cylindrical specimen was concentrically situated between two steel punches (25 mm diameter each) and vertically loaded at a 25.4 mm/min loading-rate till failure. For each mixture, three specimens were tested, and then the average of their recorded failure load values was used in computing the punching strength $\left(\sigma_{t}\right)$ as presented in Equation (5).

(5) $$\sigma_{t}=\frac{P}{\pi \left(1.2\mathrm{bt}-a^{2}\right)}$$

where P is failure load (kg), b is specimen radius (cm), t is specimen height (cm), and a is steel punch radius (cm).

- Rutting resistance of mixtures:

The static creep test, a simple and effective test to appraise the resistance to permanent deformation (rutting) of various AC mixtures, was conducted [58,59]. By conducting this testing technique, the elastic (recoverable) and plastic (irrecoverable) deformations of AC mixtures can be captured [60]. Using a Universal Testing Machine (UTM), cylindrical specimens were subjected to a uniaxial static load for 1 h followed by unloading for 30 min while recording the specimen’s vertical deformation with time. The static creep test was performed on three replicates for each asphalt mixture according to the following conditions:

Four creep aspects were considered as essential indictors of AC mixtures’ rutting tendency: (i) accumulated axial strain, (ii) creep stiffness modulus, (iii) creep compliance, and iv) creep compliance parameters [61]. The accumulated axial strain (Ɛ(t)), at any instant of time (t), is the ratio between the recorded specimen’s deformation (Δh) under axial loading and its original height (h_o_), as presented in Equation (6). Moreover, the creep stiffness modulus (S_max_) can be defined as the ratio of the applied stress (σ) to the specimen’s maximum axial strain (Ɛ_max_), as expressed in Equation (7). Therefore, the creep stiffness modulus (S_max_) value reflects the material’s ability to resist deflections caused by the applied axial load. On the other hand, the creep compliance is a fundamental characteristic of the AC mixture, as a viscoelastic material, which enlightens the relationship between the applied stress and the time-dependent strain under the applied axial load. The creep compliance J(t), at any instant of time (t) during loading, can be computed by dividing the specimen’s strain (Ɛ(t)) by the applied stress (σ), as presented in Equation (8).

(6) $$\varepsilon \left(t\right)=\frac{∆h}{h_{o}}$$

(7) $$S_{\max}=\frac{\sigma }{\varepsilon_{\max}}$$

(8) $$J\left(t\right)=\frac{\varepsilon \left(t\right)}{\sigma }$$

Moreover, creep compliance parameters (a and m) are considered the material’s regression-coefficients that identify the linear part of the material’s log creep compliance—log time curve during the loading period, in which a and m are the intercept and the slope of the curve, respectively. Usually, for estimating the creep compliance parameters, a power model is utilized, as described in Equation (9) [60,62].

(9) $$J^{\prime }=J\left(t\right)-J_{o}={\mathrm{at}}^{m}$$

where J′ is the viscoelastic component of the creep compliance at any loading time (t); J(t) is creep compliance at any loading time (t); J_o_ is the instantaneous creep compliance, which is the creep compliance at the beginning of the static creep test (usually at the time of 0.1 s); a and m are creep compliance parameters.

## 3. Experimental Results and Discussions

### 3.1. Binder Evaluation Results

#### 3.1.1. Physical Characteristics

The effects of NC particles on the physical and mechanical properties (penetration depth, softening point, penetration index, and Rotational Viscosity (RV)) of the modified asphalt binders were assessed, and all asphalt binders’ results are presented in Table 6.

As can be noted from Table 6, all modified binders exhibited lower penetration depth and higher softening point, PI, and RV compared with the base one. Moreover, incorporating NC particles further enhanced HDPE-modified binder performance, where increasing NC particle content reduced the penetration depth and increased the softening point, PI, and RV values. This reveals an enhancement in binder thermal sensitivity and stiffness, resulting in higher permanent deformation resistance at higher temperatures. This might be due to the large surface area and reactivity of the NC particles. 

#### 3.1.2. Storage Stability Results

**Figure 5:**
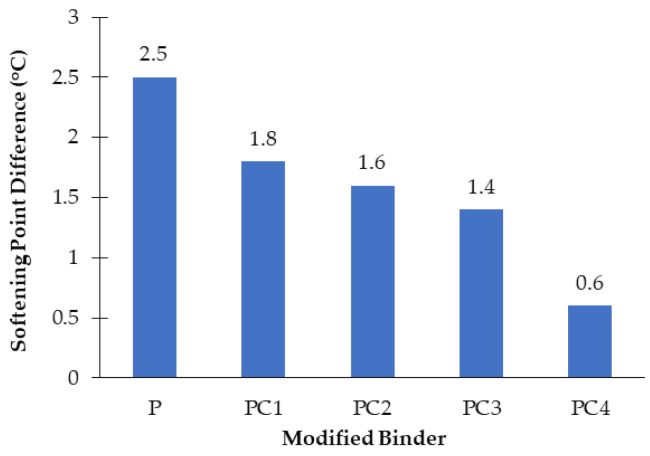
Difference in softening point between top and bottom of all modified binders.

The stability of storage testing results of HDPE –– and NC/HDPE-modified binders, based on softening point difference, are presented in Figure 5. It can be noted that S_Pdiff_ values for all modified binders are less than 2.5 °C, which implies that all modified binders exhibited acceptable storage stability. Moreover, increasing the content of NC particles caused an increase in SP_diff_ values that reveals better stability of storage level. Therefore, integrating NC particles improved the storage stability of the HDPE-modified binder through the stabilization of HDPE in asphalt binders due to its anti-separation and anti-oxidation effects. Moreover, PC4 attained superior stability of storage as its SP_diff_ value is less than 1 °C (0.6 °C).

#### 3.1.3. Fourier Transform Infrared Spectroscopy (FTIR) Results 

**Figure 6:**
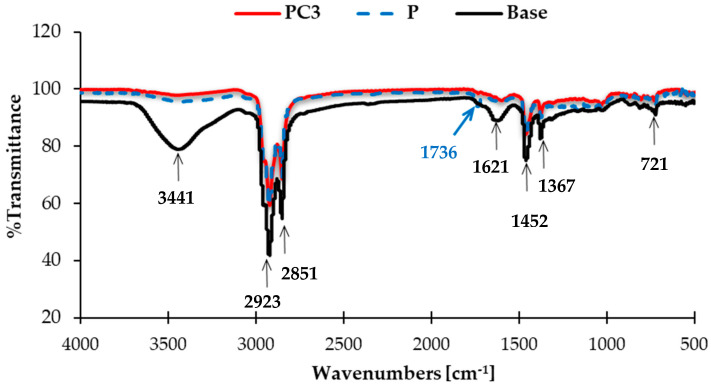
Infrared spectra of base, HDPE-, and NC/HDPE-modified asphalt.

FTIR spectroscopy was utilized to investigate chemical and structural modification in the asphalt binders due to the addition of modifiers. The resulting infrared spectra of base asphalt (base), HDPE-modified asphalt (P), and NC/HDPE-modified asphalt (PC3) are depicted in Figure 6. The assigned main bands of the FTIR spectra are listed in Table 7.

In the infrared spectra portrayed in Figure 6, the broad peak at around 3441 cm^−1^, attributed to O–H stretching of phenols, clearly appears in the base asphalt, and has almost vanished in both modified asphalts (P and PC3). This reveals an asphalt chemical interaction with modifiers, which possibly occurred via ether formation reaction. Moreover, other peaks that appeared in the base asphalt IR spectrum were conserved in both HDPE- and NC/HDPE-modified asphalt, such as peaks at 2923 and 2851 cm^−1^, 1621 cm^−1^, 1452 cm^−1^, 1376 cm^−1^, and 721 cm^−1^ that were assigned to C–H stretching, C=C stretching alkene, C–H bending of CH_2_, C–H bending of CH_3_, and C–H bending aromatic bonds, respectively. This confirms that the main hydrocarbon structure of asphalt base was maintained upon the addition of the modifier.

In the HDPE-modified asphalt IR spectrum, a new peak appeared at 1736 cm^−1^ that might represent carbonyl group (C=O) stretching frequency compared with the base asphalt IR spectrum (blue arrow). This can be correlated to an oxidation of either HDPE or some components of asphalt in the HDPE-modified asphalt [63]. Generally, asphalt oxidation is produced by a chemical change in the asphalt structure [64]. On the other hand, incorporating NC into HDPE-modified asphalt resulted in the disappearance of such C=O stretching at around 1736 cm^−1^. This may prevent the oxidation reaction that might occur upon the addition of HDPE to the base asphalt. Therefore, the addition of NC had a positive effect on maintaining the stability of the base asphalt in the HDPE-modified asphalt, which agrees with other researchers’ findings [65].

In summary, the previous qualitative analysis of the infrared spectra reveals the occurrence of one or more chemical reactions between the asphalt and modifiers (HDPE and NC). However, incorporating NC particles into the HDPE-modified asphalt had a positive effect in terms of anti-oxidation improvement.

#### 3.1.4. Scanning Electron Microscope (SEM) Results

**Figure 7:**
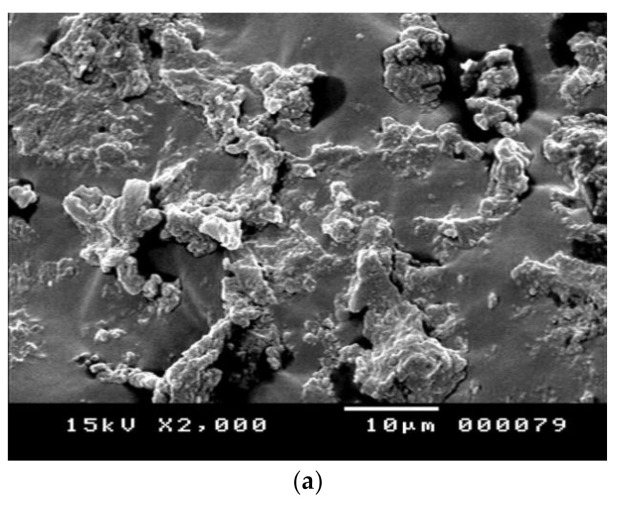

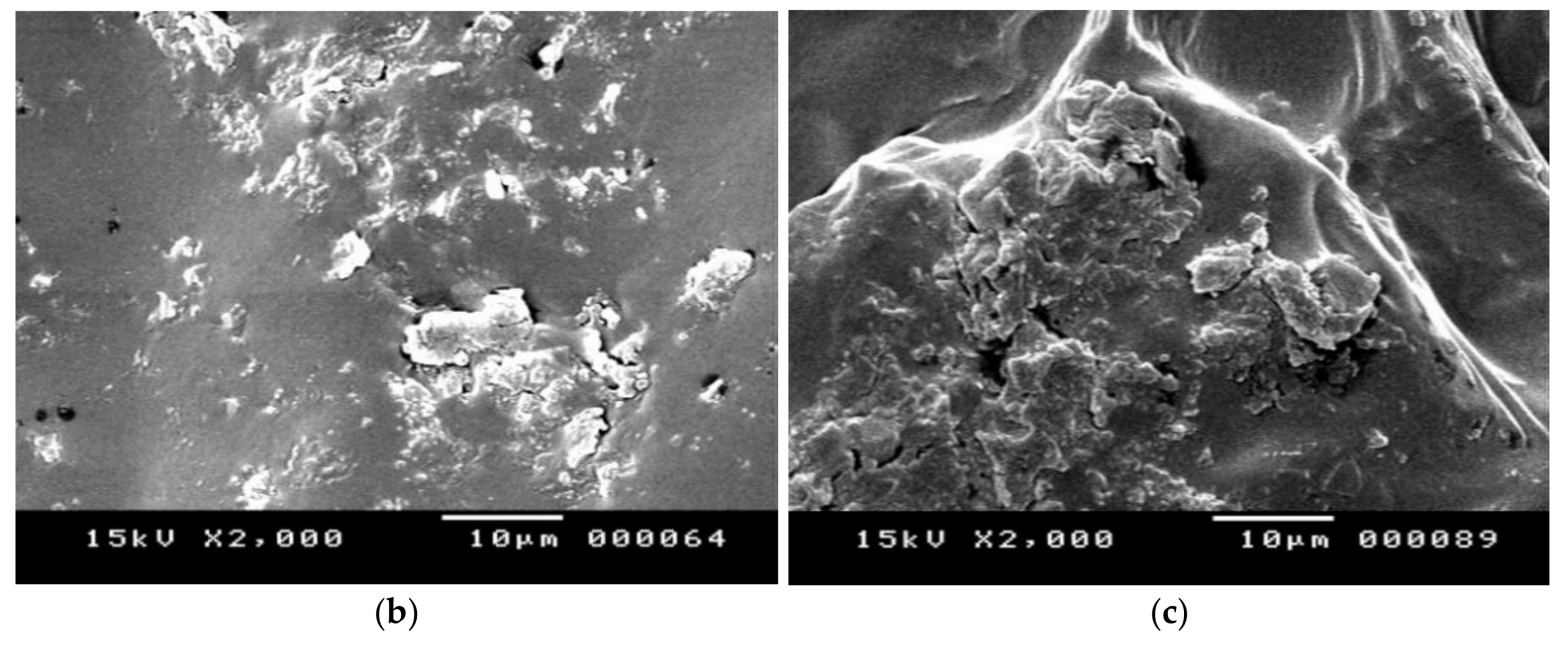
SEM micrographs of binders: (**a**) base, (**b**) HDPE-modified, and (**c**) NC/HDPE-modified.

A scanning electron microscope (SEM) was utilized to investigate the binder’s homogeneity and capture the quality of dispersion of modifiers within it. SEM micrographs of the base, HDPE-, and NC/HDPE-modified asphalt binders’ microstructural morphology are presented in Figure 7a–c, respectively. The base asphalt micrograph implies its homogeneity, as shown in Figure 7a. Moreover, additives are well distributed in the asphalt binder.

### 3.2. Mixture Evaluation Results

#### 3.2.1. Stiffness Results

**Figure 8:**
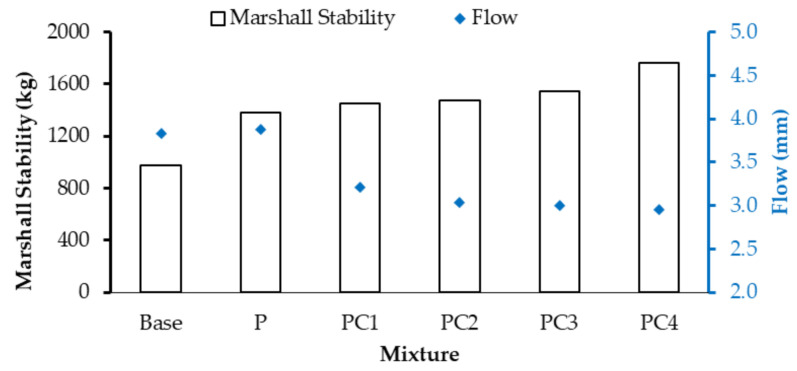
Marshall stability and flow values for conventional and modified AC mixtures.

For AC mixture stiffness (rigidity) investigation, Marshall Quotient (MQ) values were calculated based on Marshall testing results (stability and flow) (ASTM D 6927). Marshall stability and flow values for the conventional and modified AC mixtures are depicted in Figure 8. All AC mixtures satisfied Marshall stability and flow specification criteria of more than 900 kg and (2–4) mm, respectively, according to Egyptian standard specifications [47]. While the addition of HDPE increased the base AC mixture stability value (by 41.3%), no significant change was noted in its flow value, which agrees with other research efforts [66]. On the other hand, incorporating a small percentage of NC particles (1, 2, and 3% by weight of the base binder) did not improve the stability value but significantly reduced the flow value, compared with the HDPE-modified (P) mixture. However, the addition of 4% of NC (PC4) enhanced both the stability and flow values; it increased the stability value by 27.3% and reduced the flow value by 22.8%, compared with the P mixture. 

**Figure 9:**
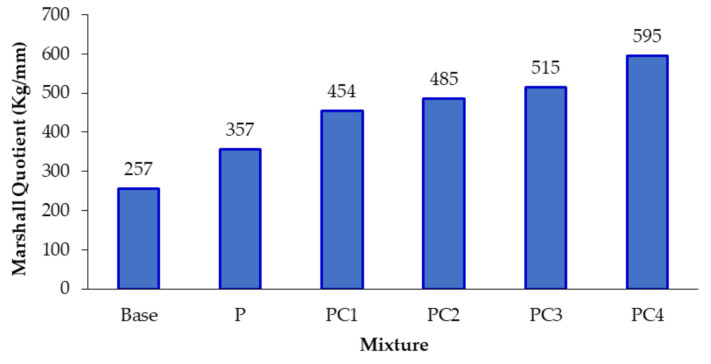
Marshall Quotient (MQ) values for conventional and modified AC mixtures.

The Marshall Quotient (MQ) values of the conventional and modified mixtures are depicted in Figure 9. An increasing trend in the MQ values was observed. Both HDPE- and NC/HDPE-modified mixtures attained higher MQ values than the conventional one. Moreover, with increasing NC content, the MQ value increased. PC4 exhibited the highest MQ value, which is about 1.3 and 0.7 times the values of the conventional (base) and HDPE-modified (P) mixtures, respectively. Therefore, incorporating NC particles into the HDPE-modified binder had a significant positive effect on increasing the mixture stiffness.

#### 3.2.2. Moisture Damage Resistance Results 

An immersion–compression test was conducted in accordance with ASTM D 1075 for measuring the reduction in the AC mixture’s compressive strength due to the moisture action. The retained strength index (IRS) was determined as the compressive strength ratio of the immersed samples to the dry ones. The IRS value is considered as a key indicator of the AC mixture’s moisture susceptibility (ASTM D 1075). 

**Figure 10:**
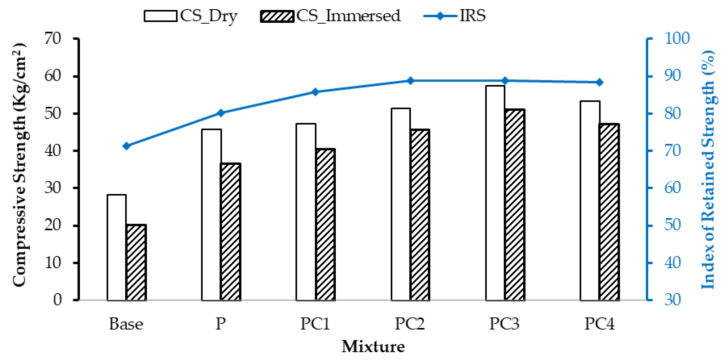
Moisture damage resistance of conventional and modified AC mixtures.

The compressive strength (dry and immersed) and IRS values for conventional and modified mixtures are depicted in Figure 10. As can be noted, all immersed samples were observed to attain lower compressive strength values compared to the dry ones. This can be attributed to the possible loss of aggregate/binder adhesion due to the water infiltration action (moisture susceptibility). However, small compressive strength differences were noted between immerged and dry samples for the NC/HDPE mixtures (PC1, PC2, PC3, and PC4). This implies that incorporating NC particles into the HDPE-modified mixtures had a positive impact on improving aggregate/binder adhesion. 

Based on the results in Figure 10, all modified mixtures attained higher compressive strength (dry and immersed) and IRS values than the conventional mixture (base). The dry compressive strength (CS _Dry_) of the base mixture increased by 61.5%, 66.7%, 81.2%, 102.7%, and 88.4% in the case of P-, PC1-, PC2-, PC3-, and PC4-modified mixtures, respectively. For the immersed compressive strength (CS _Immersed_), higher increments were noted, 81.2%, 100.1%, 125.5%, 152.3%, and 133.6%, for the above-mentioned mixtures than the base mixture. This reveals that incorporating modifiers enhanced the asphalt mixture adhesion strength and, therefore, significantly improved the immersed compressive strength. Moreover, the addition of NC particles enhanced the compressive strength of the HDPE-modified mixture under both dry and immersed conditions, in which the addition of 3% of NC (PC3) increased dry and immersed compressive strength of the HDPE-modified mixture by 25.5% and 39.2%, respectively. 

As the IRS value is considered an indicator of the asphalt mixture’s resistance against moisture, higher IRS values of modified mixtures compared with the conventional one reveal the importance of incorporating additives. The IRS value increased by 12.2%, 20.0%, 24.4%, 24.5%, and 23.9% for P-, PC1-, PC2-, PC3-, and PC4-modified mixtures, respectively, compared with the base one. Moreover, the addition of NC particles improved the moisture damage resistance of the HDPE-modified mixtures. The greatest improvement in the moisture damage resistance was observed when incorporating 3% of NC into the HDPE-modified mixture (PC3). Furthermore, IRS values for all mixtures (conventional and modified) satisfied the specification requirements (higher than 70%), as shown in Figure 10.

Moreover, the enhancement in the compressive strength (dry and immersed) and moisture damage resistance when incorporating NC can be attributed to the large surface area associated with NC particles that thereby significantly enhanced the aggregate/binder adhesion and consequently improved water damage resistance [65,67].

#### 3.2.3. Stripping Susceptibility Results

**Figure 11:**
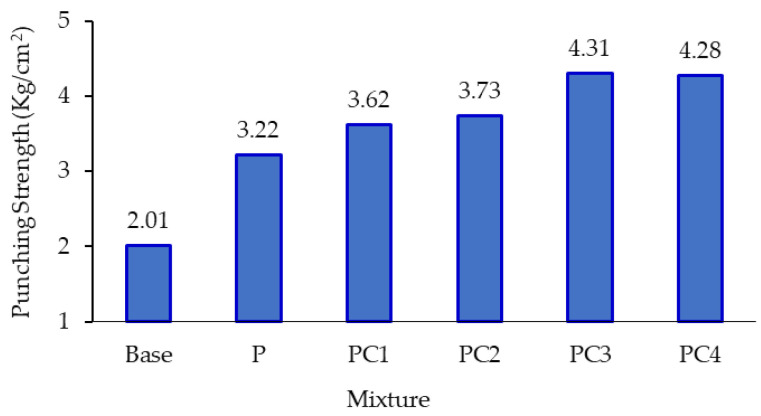
Punching strength values of AC mixtures.

The double punching (DP) test is a viable test method for quantifying the asphalt mixture’s stripping susceptibility, in which the resulting punching strength reflects the stripping behaviour between aggregate and binder in the mixture that correlates with the physical and chemical properties of both aggregate and binder [56,57]. Figure 11 presents the punching strength values of the conventional and modified asphalt mixtures. As can be noted, all modified mixtures outperformed the conventional mixture (base) in terms of the punching strength, especially when incorporating NC particles. The punching strength value for P-, PC1-, PC2-, PC3-, and PC4-modified mixtures was higher than the base mixture by 60.2%, 80.1%, 85.7%, 114.2%, and 112.8%, respectively. Moreover, the addition of NC positively enhanced the HDPE-modified mixture’s punching strength, and the highest enhancement was observed with the addition of 3% of NC (PC3), with an increment of 33.7%. This might be explained by the fact that adding NC particles enhanced the binder’s stiffness, which consequently caused an enhancement in the bonding properties of the asphalt mixture and, therefore, the punching strength [68].

#### 3.2.4. Rutting Resistance Results

Throughout the static creep test, the asphalt specimen’s vertical deformations with time (during loading and unloading) were recorded. For each AC mixture, the average of three replicates (specimens) was calculated. Then, to investigate the rutting tendency of conventional and modified AC mixtures, four creep features were considered: (i) accumulated axial strain, (ii) creep stiffness modulus, (iii) creep compliance, and (iv) creep compliance parameters.

**Figure 12:**
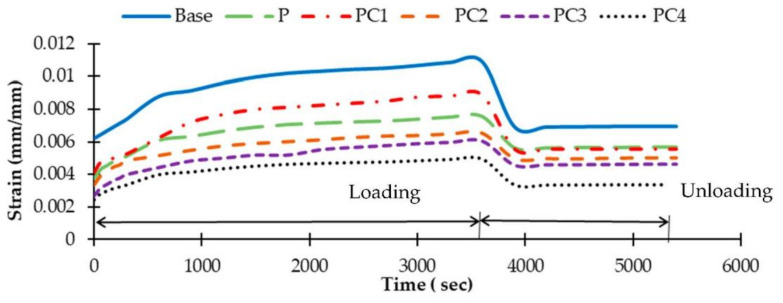
The accumulated axial strain with time for AC mixtures.

The accumulated axial strains with time, during both loading and unloading, were calculated for the conventional and modified mixtures and depicted in Figure 12. It is clear from Figure 12 that the addition of either HDPE alone or NC particles with HDPE reduced the mixture’s axial stain at different levels and hence enhanced its strength against deformation relative to the conventional mixture. However, axial stains for NC/HDPE-modified mixtures were lower than the HDPE-modified. 

From Figure 12, some criteria that characterize the strain–time diagram and identify mixtures’ elastic and plastic deformations can be drawn. These criteria include maximum stain (Ɛ_max_), which is the strain at the end of loading-period; permanent stain (Ɛ_perm_), which is the remaining strain at the end of unloading-period, and it is a vital predictor of rutting tendency [69]; and elastic strain (Ɛ_elas_), which is the neat difference between the maximum and permanent strains. These strain values for all mixtures are summarized in Table 8. Among all AC mixtures, the conventional mixture (base) attained the highest strain values, and PC4 attained the lowest values, as presented in Table 8. This implies that adding NC particles to the HDPE-modified binder improved both elastic (recoverable) and plastic (unrecoverable) AC mixture deformations. This improvement can be related to the effect of NC particles on increasing binder viscosity and stiffness.

Creep stiffness modulus (S_max_) values for all mixtures are presented in Table 8. As S_max_ reflects the material’s resistance to deflections, a higher S_max_ value implies higher rutting resistance. As can be noted from Table 8, the PC4 had the highest S_max_ and, therefore, the highest resistance to rutting. However, the unmodified mixture (base) had the lowest S_max_ and hence the highest rutting tendency. Comparing HDPE and NC/HDPE-modified mixtures, the second had higher S_max_ values (i.e., higher rutting resistance) except for the PC1.

**Figure 13:**
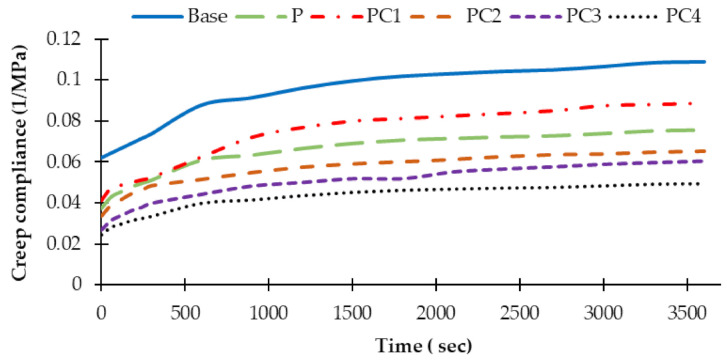
The accumulated creep compliances with time for AC mixtures.

For characterizing the viscoelastic behaviour of the used AC mixtures, two creep characterization criteria during the loading-period were estimated: creep compliances and creep compliances parameters. AC mixtures creep compliances with time during the loading-period are portrayed in Figure 13. Moreover, creep compliance parameters were estimated by applying the power low, as presented in Equation (8). Table 9 presents instantons creep compliance (J_0_) as well as creep compliance parameter (a and m) values for all used mixtures. Higher creep compliance and creep compliance parameter values reveal higher rutting potential. Therefore, modified mixtures with HDPE alone or with NC/HDPE clearly improved the rutting tendency of the mixtures compared to the unmodified one (base). Among modified mixtures, PC4 achieved the lowest creep compliance–time trend and creep compliance parameter values as well, i.e., had the lowest rutting tendency.

### 3.3. Statistical Analysis of the Experimental Results 

A statistical analysis was conducted using Kruskal–Wallis and Dunn tests to test whether incorporating NC particles significantly affected the performance of the HDPE-modified binder and mixture or not. The Kruskal–Wallis test is a nonparametric test counterpart of the analysis of variance (ANOVA) test, which is applicable in the case of uncertainty of the normality of data distribution and/or inadequate sample size [70]. As indicated early in the methods section, experimental tests were performed on three replicates for each AC binder and mixture. This means a total of 18 samples (3 replicates * 6 groups) were utilized for each experimental test. Therefore, the Kruskal–Wallis test was applicable in our statistical investigation. The Dunn test followed the Kruskal–Wallis test for performing multiple pairwise comparisons [71]. The statistical analysis was performed using GraphPad Prism version-9 [72]. A sample of an output of the utilized statistical analysis is presented in Table 10. Pairwise comparisons between HDPE- and NC/HDPE-modified binders and mixtures are presented in Table 11 and Table 12, respectively, in which significant values are highlighted. It can be noted from Table 11 that incorporating 4% of NC into the HDPE-modified binder (PC4) significantly improved the binder’s physical characteristics (Pen, SP, and RV). Moreover, the addition of 3% or 4% of NC into the HDPE-modified binder (PC3 and PC4) significantly improved the HDPE-modified binder’s temperature sensitivity and storage stabilities (PI and SP_diff_). From Table 12, incorporating 3% or 4% of NC into the HDPE-modified mixture (PC3 and PC4) significantly improved the HDPE-modified mixture’s pseudo-stiffness and stripping susceptibility (MQ and σ_t_). Moreover, a significant enhancement in the HDPE-modified mixture’s compressive strength and stripping susceptibility (CS _Dry_, CS _Immersed_, and IRS) in the case of incorporating 3% of NC was achieved, as shown in Table 12. In addition, a significant improvement was attained in both elastic (Ɛ_elas_) and plastic (Ɛ_perm_) deformations of the HDPE-modified mixture with the addition of 4% of NC.

## 4. Conclusions 

Results showed that employing NC/HDPE significantly increased binder penetration index and thereby enhanced binder temperature sensitivity. Moreover, it improved the storage stability of the HDPE-modified binder through the stabilization of HDPE within asphalt binders owing to its anti-separation and anti-oxidation effects. Moreover, NC’s large surface area significantly caused an enhancement in the aggregate/binder adhesion and thereby improved the AC mixture’s resistance against water damage and stripping. Based on static creep test results, integrating NC particles with the HDPE-modified binder not only enhanced elastic (recoverable) deformations but also plastic (unrecoverable) deformations of AC mixtures.

To this end, incorporating NC particles into HDPE-modified asphalt played an important dual-role: unified the polymer and asphalt, magnified the polymer’s effect on enhancing the asphalt performance, and consequently improved the overall AC mixture performance. The most satisfactory performance was attained when incorporating 3% of NC particles with HDPE-modified asphalt.

## Figures and Tables

**Table 1:** Summary of polymer-nanocomposite literature studies.

Nanomaterial Content (%)	Polymer Content (%)	Binder Type	Binder/AC Mixture Performance													Ref.
			P	S	V	D	ST	MS	ITS	TSR	R	SM	F	Mr	TS	
(0–3.25%) OMMT	5% SBS	85/100	↘	↗	↗	↗	↗				↗				↘	[25]
(0–2%) SiO_2_	5% SBS	60/70	↘	↗		→	↗		↗	↗				↗		[35]
(0–4%) NS	(5–7%) SBS	PG-76								↗				↗		[5]
1.5% (SBS/OMMT = 100/25)		PG 58-10	↘	↗	↑	↘	↗		↗	↗	↗			↗	↘	[21]
(3–6%) (SEBS/MMNC = 100/10)		AC-20	↘	↗	↗		↗									[30]
(2–6%) (6/4, 7/3 SBR/NC)		60/70					↗		↗	↗						[29]
(2–6%) NC	(2–6%) SBS	60/70							↑				↗	↑	↓	[6]
(0–1%) SiO_2_	(3–5%) SBR + (1–3%) PE	AH-70		↗	↑	↘								↗		[14]
(0–3%) CNT	6% SBS	PG 58-16					↗				↗					[36]
3% OMMT	(1–5%) SBS	PG 64-22									↗		→			[27]
(1–4%) NS	4% PP	80/100							↗			↗	↗			[7]
(1–7%) NC	(1–7%) EVA	AH-70	↓	↗	↑						↗				↘	[32]
(3–7%) CaCO_3_	4% SBR	60/80									↗	→		↗		[43]
(0.5–2%) (NC/HDPE/EVA = 5/10/85)		PG 64-22	↘	↗	↗						↗					[40]
(0.5–5%) NC	(1–10%) SBS	60/70	↘	↗	↗	↘		↗			↗				↘	[23]
(4–8%) NS	5% SBS	60/70	↘	↗									↑			[44]
(0–3%) Nano-CaCO_3_	(2–10%) (WPE/SBS = 40/60)	10.1 dmm	↘	→		↘					→					[45]
2%NC	4% SBS	60/70	↘	→	↗		↗				→		→			[22]
(2–6%) (SEPS/OMMT = 100/25)		60/70	↘	↗	↑		↗				↗				↘	[21]
(4–6%) NC + (4–6%) Nano Lime	3% SBS	PG 64-22					↗				↗				↘	[38]
(0–6%) NC + (0–6%) NanoLime	3% SBS	PG 64-22			↗						↑					[39]
(0.6–3%) Nano-Al_2_O_3_	(3–5%) SBS	60/70			↗		↗				↗					[37]
3% MMT	6% SBS or 7% SBR	60/70	↘	↗	↗		↗				→		→			[28]
(0–5%) MMT	5% SBS	60/70	↘	↗	↗	↘		↗			↗			↗	↘	[24]

AC= asphalt concrete; P = penetration; S = softening point; V = viscosity; D = ductility; ST = storage stability; MS = Marshall stability; ITS = indirect tensile strength; TSR = tensile strength ratio; R = rutting parameter; SM = stiffness modulus; F = fatigue parameters; Mr = resilient modulus; TS = temperature sensitivity; SBS = styrene-butadiene-styrene; SBR = styrene-butadiene-rubber; SEBS = styrene-ethylene/butylene-styrene; EVA = ethyl-vinyl-acetate; PP = polypropylene; PE = polyethylene; HDPE = high-density polyethylene; NC = nanoclay; MMT = Montmorillonite clay; OMMT = organically modified Montmorillonite clay; NS = nanosilica; CNT = carbon-nanotubes. ↑ = significant increase; ↗ = slight increase; → = no significant change; ↘ = slight decrease; ↓ = significant decrease.

**Table 2:** Physical and mechanical characteristics of the base asphalt binder.

Test	Standard	Value
Penetration (100 g, 25 °C, 5 s), dmm	ASTM D5	62
Ductility (25 °C, 5 cm/min), cm	ASTM D113	100
Softening Point, °C	ASTM D36	45.4
Rotational viscosity at 135 °C, C.st	ASTM D 2170	376
Flash point, °C	ASTM D92	250
Density at 15 °C, g/cm^3^	ASTM D70	1.01
Penetration index (PI)		−1.95

**Table 3:** Physical and mechanical properties data sheet of HDPE.

Property	Standard	Value
Density, g/cm³	ASTM D4883	0.956
Melt Index (190 °C/2.16 kg), g/10 min	ASTM D1238	20
Peak Melting Temperature, °C	ASTM D3418	130
Tensile Stress at Yield, MPa	ISO 527-2/1A/50	23
Tensile Strain at Yield, %	ISO 527-2/1A/50	10
Tensile Strain at Break, %	ISO 527-2/1A/50	>100
Flexural Modulus, MPa	ISO 178	920
Notched Izod Impact Strength, kJ/m²	ISO 180/1A	4.3

**Table 4:** Physical and chemical properties of NC.

Property	Value
Physical state	Powder
Colour	White
Shape	Spherical
Size, nm	<40
Compound name	Aluminium Silicate Hydroxide
Chemical formula	Al_2_Si_2_O_5_(OH)_4_
Purity, %	>99
Specific gravity, g/cm³	2.6
Molecular Weight, g/mole	258.2
Melting Point, °C	>1500

**Table 5:** Marshall design parameters for the conventional AC mixture.

Marshall Parameter	Value	Specification ^1^	
		Min.	Max.
Bulk density, t/m^3^	2.35	-	-
Stability, kg	979.5	900	-
Flow, mm	3.83	2	4
Air voids (AV), %	4.32	3	5
Voids in mineral aggregates (VMA), %	15.83	13	-
Voids filled with binder (VFB), %	72.7	70	-
Optimum asphalt content (OAC), %	5	-	-

^1^ according to the Egyptian Highway standard.

**Table 6:** Physical characteristics of base, HDPE-, and NC/HDPE-modified binders.

Binder	Penetration (dmm)	Softening Point (°C)	Penetration Index	Rotational Viscosity at 135 °C (C.st)
Base	62.0	45.4	− 1.95	376.0
P	38.7	60.0	0.41	1966.7
PC1	36.0	62.0	0.63	2239.3
PC2	30.0	65.0	0.78	2478.0
PC3	24.7	67.4	0.78	2587.0
PC4	22.3	69.0	0.84	2701.0

**Table 7:** The assigned main bands of the FTIR spectra [63,64,65].

Band Position (cm^−1^)	Assignation
3441	O–H stretching phenols
2923, 2851	C–H asymmetric stretching
1736	C=O stretching
1621	C=C stretching alkene
1452	C–H bending of -(CH2)n-
1376	C–H bending of CH3
721	C–H bending aromatic

**Table 8:** Creep strain properties and stiffness modulus for AC mixtures.

AC Mixture	Stain Properties			Stiffness Modulus (S_max_)
	Maximum Stain (Ɛ_max_)	Permanent Stain (Ɛ_perm_)	Elastic Strain (Ɛ_elas_)	
Base	0.0098	0.0062	0.0036	9.18
P	0.0076	0.0057	0.0019	13.23
PC1	0.0089	0.0056	0.0033	11.27
PC2	0.0065	0.0050	0.0015	15.31
PC3	0.0060	0.0046	0.0014	16.5
PC4	0.0049	0.0034	0.0016	20.24

**Table 9:** Creep compliance parameters during loading period.

AC Mixture	Instantaneous Creep Compliance (J_0_)	Creep Compliance Parameters	
		a	m
Base	0.0621	0.0010	0.4886
P	0.0374	0.0009	0.4689
PC1	0.0413	0.0005	0.5673
PC2	0.0336	0.0013	0.4049
PC3	0.0269	0.0008	0.4616
PC4	0.0244	0.0006	0.4689

**Table 10:** A sample of the statistical analysis output.

**Kruskal–Wallis Test**						
*p*-value		0.0067				
Exact or approximate *p*-value?		Approximate				
***p*-value summary**		**				
Do the medians vary significantly? (*p* < 0.1)?		Yes				
Number of groups		5				
Kruskal-Wallis statistic		16.06				
**Data summary**						
Number of treatments (columns)		5				
Number of values (total)		15				
Number of families		1				
Number of comparisons per family		4				
Alpha		0.1				
**Dunn’s multiple comparisons test**	Mean rank difference	Significant?	Summary	Adjusted *p* Value	B-?	
P (C) vs. PC1	−2.333	No	Ns	<0.9999	A	PC1
P (C) vs. PC2	−6.333	No	Ns	0.73	C	PC2
P (C) vs. PC3	−11.67	Yes	*	0.04	D	PC3
P (C) vs. PC4	−8	No	Ns	0.33	E	PC4
**Test details**	Mean rank 1	Mean rank 2	Mean rank diff.	n1	n2	Z
P (C) vs. PC1	5.333	7.667	−2.333	3	3	0.5353
P (C) vs. PC2	5.333	11.67	−6.333	3	3	1.453
P (C) vs. PC3	5.333	17	−11.67	3	3	2.677
P (C) vs. PC4	5.333	13.33	−8	3	3	1.835

**Table 11:** P-values for pairwise comparisons of performance characteristics of binders.

Test Details	Binder Performance Characteristics				
	Pen	SP	PI	RV	SP_diff_
P vs. PC1	>0.99	>0.99	>0.99	>0.99	>0.99
P vs. PC2	0.84	>0.99	0.77	0.84	0.19
P vs. PC3	0.19	0.18	0.07	0.19	0.06
P vs. PC4	0.03	0.08	0.05	0.03	0.02

**Table 12:** *p*-values for pairwise comparisons of performance characteristics of mixtures.

Test Details	AC-Modified Mixture Performance Characteristics										
	MQ	CS _Dry_	CS _Immersed_	IRS	$\sigma_{t}$	Ɛ_max_	Ɛ_perm_	Ɛ_elas_	S_max_	a	b
P vs. PC1	>0.99	>0.99	>0.99	>0.99	>0.99	>0.99	>0.99	>0.99	>0.99	0.30	0.19
P vs. PC2	0.84	>0.99	0.73	0.39	>0.99	>0.99	>0.99	0.76	>0.99	>0.99	>0.99
P vs. PC3	0.09	0.06	0.04	0.05	0.07	0.84	0.32	0.26	0.83	>0.99	>0.99
P vs. PC4	0.03	0.53	0.33	0.63	0.09	0.19	0.05	0.09	0.17	>0.99	>0.99
